# Preparation and Properties of Wood Tar-based Rejuvenated Asphalt

**DOI:** 10.3390/ma13051123

**Published:** 2020-03-03

**Authors:** Xuefei Zhang, Juncai Zhu, Chaofan Wu, Qingding Wu, Kefei Liu, Kang Jiang

**Affiliations:** 1 School of Civil Engineering, Central South University of Forestry & Technology, Changsha 410004, China; 20181100329@csuft.edu.cn (X.Z.); 20171100289@csuft.edu.cn (J.Z.); T20030452@csuft.edu.cn (Q.W.); 2 Hunan Provincial Engineering Research Center for Construction Solid Wastes Recycling, Changsha 410205, China; cfwu0188@126.com; 3 Hunan Communications Research Institute Co. LTD, Changsha 410015, China

**Keywords:** wood tar, rejuvenator, rejuvenated asphalt, orthogonal test, low-temperature performance, infrared spectroscopy

## Abstract

In order to explore the applicability of the rejuvenated asphalt with wood tar as the main raw material, the orthogonal test was used to determine the optimal ratio of wood tar-based rejuvenator. The physical properties, rheological properties and components of matrix asphalt, aged asphalt, wood tar-based rejuvenated asphalt and commercial RA-102# rejuvenated asphalt were tested and compared. The results show that the optimal ratio of wood tar-based rejuvenator is 15wt% wood tar, 0.3wt% biomass fiber, 5wt% plasticizer, 0.3wt% compatibilizer, and 1wt% stabilizer of the mass of aged asphalt. Wood tar-based rejuvenator can restore the physical properties of the aged asphalt to meet the specification requirements. The synergistic effect of biomass fiber and plasticizer make the wood tar-based rejuvenated asphalt has good resistance to accumulated permanent deformation, but its low-temperature cracking resistance needs to be further improved. During the rejuvenation process of aged asphalt, the colloidal state changes from gel-state to sol-state, characterizing that the viscosity of asphalt decreased and the fluidity increased. Wood tar-based rejuvenator can react with aged asphalt to weaken the vibration strength of carbonyl and sulfoxide groups, so as to realize the recovery of service performance. Wood tar-based rejuvenator has better environmental protection and applicability, which is worthy of further study and promotion.

## 1. Introduction

Affected by the repeated action of vehicle load and natural factors (rain, snow, temperature, sunlight, etc.), asphalt pavement is easy to harden, brittle, and crack due to aging in the long-term service period, resulting in more serious rutting, cracking, loosening, and other forms of pavement diseases [1,2]. Meanwhile, lots of reclaimed asphalt pavement (RAP) will be produced during the repair and maintenance of damaged pavement, which will not only occupy the land, but also cause environmental pollution. In addition, the use of new asphalt and its mixture to replace RAP requires a great amount of resources and funds [3]. Therefore, the use of RAP can not only save resources and reduce the cost of asphalt pavement, but also decrease the environmental pollution caused by waste asphalt materials, which has significant economic, social and environmental benefits [4,5].

The key of RAP rejuvenation technology is to realize the rejuvenation of aged asphalt and restore its road performance [6]. At present, the rejuvenation of aged asphalt mainly supplies the volatile and light molecular components lost in the process of aging by adding rejuvenator containing a large number of light components [7,8], so as to provide a uniform system for the full dissolution of asphaltenes and the prevention of precipitation or flocculation [9]. To evaluate the effect of rejuvenator, many scholars used conventional testing, rotatory viscometer (RV), dynamic shear rheometer (DSR), and bending beam rheometer (BBR) to evaluate the physical properties and rheological properties of rejuvenated asphalts [10,11,12]. Moreover, the colloidal structure, intermolecular force and the changes of chemical functional groups of rejuvenated asphalt were tested by components analysis (SARA) and Fourier infrared spectroscopy (FTIR), and the rejuvenation mechanism was revealed from the micro-scale [13]. The results showed that a proper amount of rejuvenator can soften the aged asphalt and reduce its viscosity, improve its fluidity, make its colloidal structure changes from gel-state to sol-gel state, then restore the elastic property and have similar or even better performance with the original asphalt [10].

Recently, many research institutions have developed different kinds of rejuvenators, such as composite rejuvenator mixed with aromatic rich solvent oil and resin [14], treated waste engine oil rejuvenator [15,16], industrial waste rubber rejuvenator and composite rejuvenator synthesized with different components [17]. However, many rejuvenators have the defects of high preparation cost, hidden safety hazards (due to low flash point), and some rejuvenators are prone to cause environmental pollution [18]. Therefore, it is urgent to develop new rejuvenators with low production cost, good rejuvenation effect and environmental protection [19,20,21]. With the continuous development of pyrolysis technology, the bio-oil with low viscosity, high flash point, environmental friendliness, renewable and other characteristics as the rejuvenator of aged asphalt has been widely concerned by many scholars [22]. Many studies have shown that the bio-oil rich in aromatic light components can effectively supplement the saturates and aromatics missing in aged asphalt, and the appropriate amount of bio-oil can effectively restore the road performance of aged asphalt and make it meet the requirements of specification [23,24].

According to the sources of raw materials, bio-oil is often divided into agricultural and forestry products bio-oil, animal waste bio-oil and waste bio-oil [25,26]. Zaumanis [27] studied the effect of pine oil on the rejuvenation of aged PG64-22 asphalt, and the results showed that the 25 °C viscosity of rejuvenated asphalt can be restored to the level of original asphalt, and the rejuvenated asphalt mixture has good rutting resistance and excellent low-temperature cracking resistance. Oldham [28] used RV, DSR and three point bending test to evaluate the performance of rejuvenated asphalt which is rejuvenated by swine manure extracted bio-oil. The results showed that the bio-oil extracted from swine manure can reduce the viscosity of aged asphalt, and improve its ductility, fracture energy and low-temperature cracking resistance. Lots of studies have shown that the physical and rheological properties of waste cooking oil (WCO) rejuvenated asphalt can be restored to the same level as the original asphalt. In addition, Sun’s [15] FTIR test results proved that there is no chemical reaction between WCO and aged asphalt, only physical blending. Futhermore, WCO can increase the vibration intensity of carbonyl group in aged asphalt, but it has little effect on the vibration intensity of sulfoxide group [29]. Thus, it can be seen that the rejuvenation of aged asphalt by bio-oil, which is from a wide ranges of sources and environmentally friendly, has a broad application prospect.

As a kind of bio-oil of agricultural and forestry products [30], the wood tar can be obtained by processing crop straw, bamboo, wood and other plant materials rich in cellulose, hemicellulose and lignin by pyrolysis technology, which has the characteristics of low cost, large output, green and renewable [31]. In addition, the process of producing wood tar is accompanied by the production of high value-added bio-carbon, wood gas, wood vinegar and other energy products [32]. Therefore, the preparation of wood tar can not only realize the resource recovery and harmless treatment of agricultural and forestry wastes, but also produce huge economic, environmental and social benefits [33]. Our recent work has demonstrated that the wood tar can effectively improve the physical and rheological properties of the original asphalt, and adjust the components of asphalt and react with them. Nevertheless, the research on the preparation of asphalt rejuvenation from wood tar has not been carried out or reported in depth. Based on the premise that wood tar has similar properties to the missing components in aged asphalt, we hypothesize that wood tar has the potential to rejuvenate aged asphalt, and high performance composite asphalt rejuvenator can be prepared in combination with other auxiliary agents for RAP rejuvenation. Further, to optimize the performance of wood tar-based rejuvenator, bamboo fiber, another kind of biomass material, is used as the fiber stabilizer to improve the ratio of free asphalt and structural asphalt in the rejuvenated asphalt mortar, so as to improve the service performance and durability of the rejuvenated asphalt [34].

In this paper, wood tar is used as the basic raw material to prepare the composite rejuvenator by mixing different proportions of wood tar, biomass fiber, plasticizer, and fixed proportions of compatibilizer and stabilizer. Sixteen combinations of three factors and four levels are selected by orthogonal experiment to determine the optimal dosage of wood tar, biomass fiber and plasticizer. Laboratory tests are conducted on the matrix original asphalt, aged asphalt, wood tar-based rejuvenated asphalt and commercial RA-102# rejuvenated asphalt to evaluate the physical properties, rheological properties and composition of the four kinds of asphalt to analyze the feasibility of using wood tar-based rejuvenator for RAP rejuvenation. The results will help to expand the utilization of wood tar and enrich the varieties of asphalt rejuvenators, and lay a foundation for the promotion and application of biomass based rejuvenators.

## 2. Materials and Experiments

### 2.1. Materials

#### 2.1.1. Raw Materials

The matrix asphalt is Gaofu 70# petroleum asphalt produced by Zhengcheng Petrochemical Co., Ltd. (Maoming, China). Its basic properties are shown in Table 1.

Wood tar, biomass fiber, plasticizer, stabilizer and compatibilizer are used as the basic components to prepare wood tar-based rejuvenator. The wood tar is produced in an environmental charcoal factory in Youxian County (Hunan, China), its raw material is bamboo. The basic properties of bamboo are shown in Table 2. The moisture content of bamboo-based wood tar is less than 5%, and the content of cresol is more than 10% [32]. Biomass fiber is a kind of modified flocculent fiber made from stem or bark of bamboo and wood, its length is 400~2000 μm, density is 0.91~0.95, and moisture content is less than 3%. Plasticizer (dioctyl phthalate), stabilizer (lauryl propylene diamine), and compatibilizer (maleic anhydride) were purchased from Jirui Chemical Equipment Co., Ltd. (Changsha, China), analytically pure.

For comparative study, RA-102# rejuvenator provided by Subote New Materials Co., Ltd. (Nanjing, China) is selected as reference rejuvenator, and its basic properties are shown in Table 3. It can be seen that all indexes in the table meet the requirements of Technical Specifications for Highway Asphalt Pavement Recycling (JTG F41-2008) [35].

**Figure 1:**
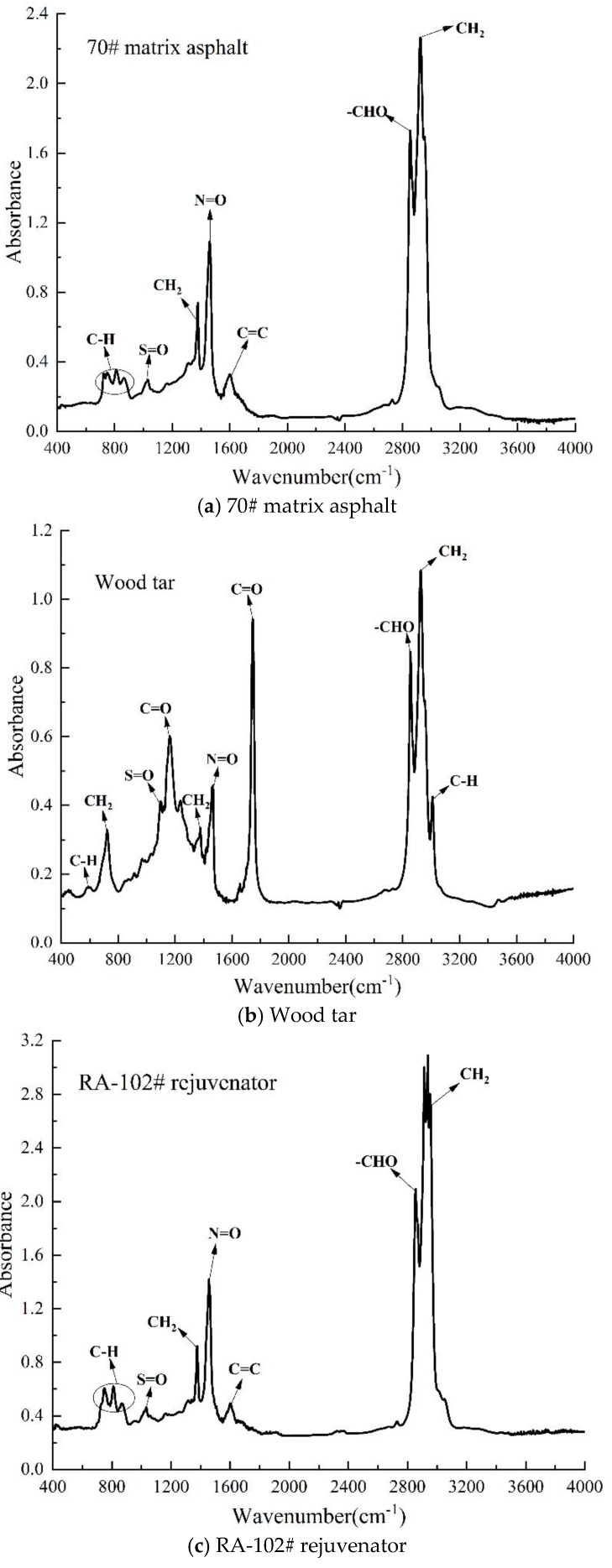
Infrared spectrum of each raw material (**a**) 70# matrix asphalt; (**b**) wood tar; (**c**) RA-102# rejuvenator.

The infrared spectra of 70# matrix asphalt, wood tar and RA-102# rejuvenator are shown in Figure 1. As can be seen from the figure that there are a large number of C-H bonds (wavenumber 720–885 cm^−1^) and N=O bonds (wavenumber 1400–1500 cm^−1^) in the matrix asphalt, so the content of light components, such as aromatic compounds, is relatively high [36,37]. The presence of C=O bond at 1760 cm^−1^, S=O bond at 1030 cm^−1^, and C-H bond at 600–800 cm^−1^ means that the wood tar contains a certain amount of carbonyl, sulfoxide, and aromatic hydrocarbons, respectively [13]. RA-102# rejuvenator has S=O bond at 1030 cm^−1^, C-H bond at 720–885 cm^−1^ and 1600 cm^−1^ and N=O bond at 1400–1500 cm^−1^, corresponding to sulfoxide, aromatic hydrocarbons and aromatic compounds, respectively. The matrix asphalt and RA-102# rejuvenator have a very similar chemical structure, which means they have similar chemical properties and good compatibility [15,38].

#### 2.1.2. Aged Asphalt

The original asphalt is subjected to thin film oven test (TFOT) and pressure aging vessel (PAV) to prepare aged asphalt. The test condition of TFOT is 163 °C aging for 5 h, and the test condition of PAV is 100 °C/2.1 MPa aging for 20 h. The effects of asphalt binder aging in construction and in service are simulated respectively [39].

#### 2.1.3. Rejuvenated Asphalt

The composition of wood tar-based rejuvenated asphalt includes aged asphalt, wood tar, biomass fiber, plasticizer, compatibilizer and stabilizer. Among them, wood tar is added at 5wt%, 10wt%, 15wt% and 20wt% of the mass of the aged asphalt; biomass fiber is added at 0.3wt%, 0.4wt%, 0.5wt% and 0.6wt% of the mass of the aged asphalt; plasticizer is added at 2wt%, 3wt%, 4wt% and 5wt% of the mass of the aged asphalt. Based on laboratory test results, the content of compatibilizer and stabilizer is 0.3wt% and 1wt% of the mass of aged asphalt, respectively.

RA-102# rejuvenated asphalt is composed of aged asphalt and 10wt% RA-102# rejuvenator.

### 2.2. Sample Preparation

**Figure 2:**
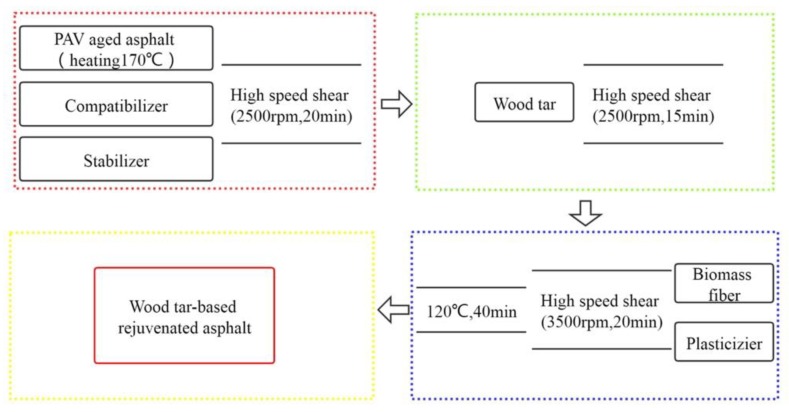
Preparation process of wood tar-based rejuvenated asphalt.

The preparation process of wood tar-based rejuvenated asphalt sample is shown in Figure 2. RA-102# rejuvenated asphalt is prepared by heating the aged asphalt to 170 °C and adding RA-102# rejuvenator, then using high-speed shear to stir at 2500 rpm for 20 min.

### 2.3. Experiments

**Figure 3:**
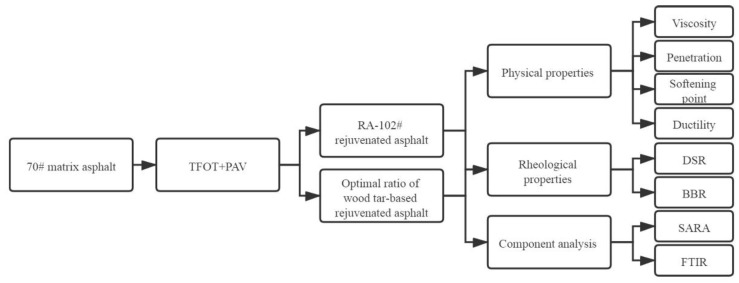
Test scheme.

The penetration, softening point, ductility and viscosity of rejuvenated asphalt are tested by orthogonal experiment to determine the optimal ratio of wood tar-based rejuvenator. The physical properties, rheological performance, and components of wood tar-based rejuvenated asphalt are tested under the optimal ratio, and compared with RA-102# rejuvenated asphalt. The test scheme is shown in Figure 3.

#### 2.3.1. Penetration

The test is conducted according to penetration of asphalt test method T0604-2011 in JTG E20-2011 [39]. Firstly, the asphalt sample is injected into a standard sample dish, cooled for 1.5 h at room temperature, and then placed in a thermostatic water bath at 25 °C ± 0.1 °C for 1.5 h. The 5-s penetration value of 100 g ± 0.05 g standard needle is the penetration value of asphalt. The higher the penetration value, the softer the asphalt [40].

#### 2.3.2. Softening Point

The test is conducted according to softening point of asphalt test method T0606-2011 in JTG E20-2011 [39]. Firstly, the sample ring and other parts containing asphalt sample are placed in a thermostatic water bath at 5 °C ± 0.5 °C for 15 min. Then place the test ring with asphalt in the correct position of the test device, and place the steel ball in the center of the locating ring. Turn on the electromagnetic oscillation agitator and heat it at the rate of 5 °C ± 0.5 °C/min. Read the current temperature when the asphalt softens and falls to contact with the bottom plate of the device, which is the softening point of the asphalt. The higher the softening point of asphalt, the better the high-temperature stability and the lower the temperature sensitivity [41].

#### 2.3.3. Ductility

The test is conducted according to ductility of asphalt test method T0605-2011 in JTG E20-2011 [39]. Firstly, pour the asphalt sample in the test mold and placed at room temperature for 1.5 h, then use a hot scraper to scrape the surface of the test mold flat. Place the sample together with the test mold in a thermostatic water bath at 15 °C for 1.5 h and then fix it at the designated position. Start the ductility meter, and the reading on the scale when the asphalt is pulled off is the ductility value of the asphalt at 15 °C. The larger the ductility value, the better the malleability of asphalt [25].

#### 2.3.4. Viscosity

Following the AASHTO T316-13 method, the rotational viscometer is employed to determine the viscosity of each asphalt at 135 °C. The lower the viscosity of asphalt, the better the fluidity and the lower the mixing cost [42]. However, too small viscosity value will affect the high-temperature performance of the asphalt mixture [43].

#### 2.3.5. Complex Modulus and Phase Angle

According to AASHTO T315-12, the complex modulus G* and phase angle *δ* of different asphalts are tested by dynamic shear rheometer (DSR). Testing asphalt samples by temperature scanning mode, the parallel plates with diameter of 25 mm and gap of 1 mm were selected for the tests. The test temperature ranges from 30 °C to 72 °C. The larger complex modulus and the smaller phase angle indicate that the asphalt has better deformation resistance and deformation recovery ability at high temperature [44].

#### 2.3.6. Creep Stiffness and Creep Rate

The low-temperature property of each asphalt is evaluated by bending beam viscometer (BBR) following the AASHTO T313-12, the test temperature are −6 °C, −12 °C, and −18 °C, respectively.

#### 2.3.7. Component Analysis (SARA)

The aging process of asphalt is also the process of its composition change [24]. The change of each component before and after asphalt aging and rejuvenation can be obtained by component separation test. The test is conducted according to chemical components of asphalt test method T0618-2011 in JTG E20-2011 [39]. Firstly, the asphaltene is obtained by dissolving asphalt in n-heptane and extracting at high temperature; then pour the remaining solution into the glass adsorption column containing alumina powder, rinse it with different solvents to obtain the saturates, aromatics and colloid in turn. The gel index *I_C_* is the ratio of the sum of the contents of asphaltene and saturates and the sum of the contents of aromatics and colloid. *I_C_* represents the fluidity of asphalt at specified temperature [45]. The greater the *I_C_* value, the worse the fluidity of asphalt, and the more inclined asphalt is to gel-state; the smaller the *I_C_* value, the better the fluidity of asphalt, and the more inclined asphalt is to sol-state [45].

#### 2.3.8. Fourier Transform Infrared Spectroscopy (FTIR)

FTIR can reveal the changes of chemical functional groups in the process of asphalt aging and rejuvenation from the micro scale, thus helping to clarify the mechanism of asphalt aging/rejuvenation [46]. The Shimazu IRAffinity-1S type infrared spectrometer is used to test the peak strength of S=O functional group at 1030 cm^−1^ and the peak strengths of six C=O groups within the range of 600–2000 cm^−1^ in the infrared spectrum of asphalt after aging/rejuvenation. The mixed solution was prepared by mass ratio of 1:20 between asphalt and carbon disulfide. After evenly oscillation, the solution was dropped onto the potassium bromide tablet and dried in a vacuum drying oven at 45 °C for 30 min, then take it out for testing.

The data analysis software Origin is used to analyze the peak changes of sulfoxide group (S=O bond) and carbonyl group (C=O bond) before and after asphalt aging/rejuvenation, and the aging coefficient *I* of each group is calculated [13]. The increase of I value indicates that the aging degree of asphalt is deepened, while the decrease of I value indicates that the aged asphalt is rejuvenated.

Where, S=O functional group is mainly distributed at 1030 cm^−1^ [15], *I*_S=O_ value can be calculated according to Formula (1). The larger the *I*_S=O_ value is, the deeper the aging degree of asphalt is, manifests that the viscosity value of asphalt increases [47].

(1) $$I_{S=O}=\frac{{\mathrm{Area}\;\mathrm{of}\;\mathrm{sulfoxide}\;\mathrm{peak}(1030\mathrm{cm}}^{-1})\;}{\mathrm{Peak}\;\mathrm{area}(\sum {600\mathrm{cm}}^{-1}{\;–\;2000\mathrm{cm}}^{-1})}$$

The C=O functional groups are divided into carboxylic acid, aldehyde, amide, anhydride, ester and ketone according to the differences in the effects of different C=O groups on the properties of asphalt [48]. The wavenumber range and vibration form of each group and its influence on asphalt are shown in Table 4 [48].

The *I* value of each group is the ratio of the area of spectrum of its corresponding wavenumber segment to the area of 600–2000 cm^−1^ spectrum [48], namely:

(2) $$I_{\mathrm{Carboxylic}\;\mathrm{acid}}=\frac{{\mathrm{Area}\;\mathrm{of}\;\mathrm{carboxylic}\;\mathrm{acid}(1700–1725\mathrm{cm}}^{-1})\;}{\mathrm{Peak}\;\mathrm{area}(\sum {600\mathrm{cm}}^{-1}{\;–\;2000\mathrm{cm}}^{-1})}$$

(3) $$I_{\mathrm{Aldehyde}}=\frac{{\mathrm{Area}\;\mathrm{of}\;\mathrm{aldehyde}(1720–1740\mathrm{cm}}^{-1})\;}{\mathrm{Peak}\;\mathrm{area}(\sum {600\mathrm{cm}}^{-1}{\;–\;2000\mathrm{cm}}^{-1})}$$

(4) $$I_{\mathrm{Amide}}=\frac{{\mathrm{Area}\;\mathrm{of}\;\mathrm{amide}(1640–1690\mathrm{cm}}^{-1})\;}{\mathrm{Peak}\;\mathrm{area}(\sum {600\mathrm{cm}}^{-1}{\;–\;2000\mathrm{cm}}^{-1})}$$

(5) $$I_{\mathrm{Anhydride}}=\frac{{\mathrm{Area}\;\mathrm{of}\;\mathrm{anhydride}(1740–1775\mathrm{cm}}^{-1}{\;\mathrm{and}\;1800–1830\mathrm{cm}}^{-1})\;}{\mathrm{Peak}\;\mathrm{area}(\sum {600\mathrm{cm}}^{-1}{\;–\;2000\mathrm{cm}}^{-1})}$$

(6) $$I_{\mathrm{Ester}\;}=\frac{{\mathrm{Area}\;\mathrm{of}\;\mathrm{ester}\;(1735–1750\mathrm{cm}}^{-1})\;}{\mathrm{Peak}\;\mathrm{area}(\sum {600\mathrm{cm}}^{-1}{\;–\;2000\mathrm{cm}}^{-1})}$$

(7) $$I_{\mathrm{Ketone}}=\frac{{\mathrm{Area}\;\mathrm{of}\;\mathrm{ketone}(1665–1715\mathrm{cm}}^{-1})\;}{\mathrm{Peak}\;\mathrm{area}(\sum {600\mathrm{cm}}^{-1}{\;–\;2000\mathrm{cm}}^{-1})}$$

**Figure 4:**
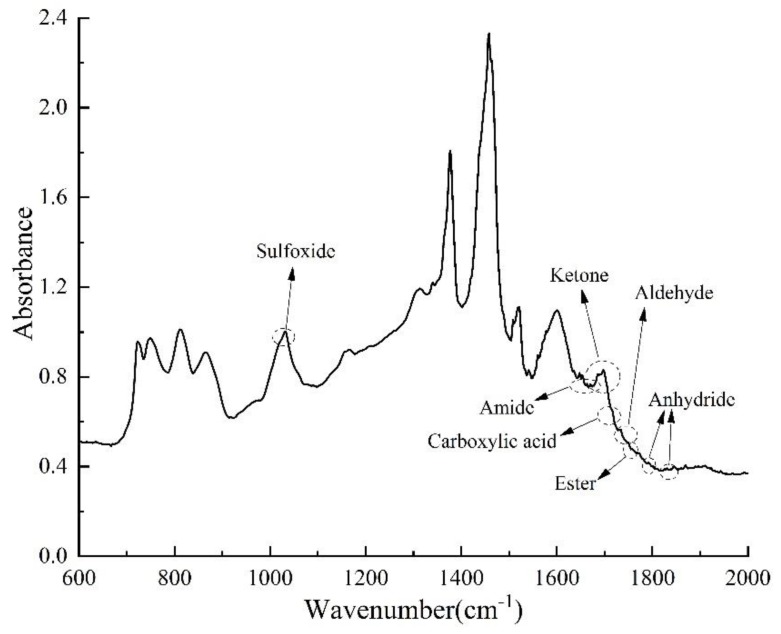
The positions of seven groups in the FTIR spectrum of aged asphalt.

The positions of seven groups in the aged asphalt are shown in Figure 4. The *I* value and its change rule of each group before and after asphalt aging/rejuvenation are calculated and analyzed respectively, and the effect of each rejuvenator is clarified [38,48].

## 3. Optimal Ratio of Wood Tar-Based Rejuvenator

### 3.1. Orthogonal Test Combination

The composition and content of raw materials of wood tar-based rejuvenator vary greatly. 64 (4^3^) samples need to be prepared if the tests of all dosages of each component are completed. To simplify the test without affecting the test results, the orthogonal test method is used to design the test to determine the optimal ratio of wood tar-based rejuvenator [53]. Taking wood tar, biomass fiber, and plasticizer as variable factors, and four change levels are taken for each variable factor. Therefore, 16 combinations are used to prepare wood tar-based rejuvenator, and the penetration, softening point, ductility and viscosity of rejuvenated asphalt are tested respectively. The wood tar, biomass fiber and plasticizer are numbered A, B, and C respectively, and the four levels are numbered 1, 2, 3 and 4 respectively. The test results are analyzed by range analysis. Range is the difference between the maximum value and the minimum value of a single performance index, which can be used to determine the most influential factor [54]. The greater the range, the greater the influence of this factor on a certain performance index. The composition and test results of each combination are shown in Table 5.

### 3.2. Penetration

**Figure 5:**
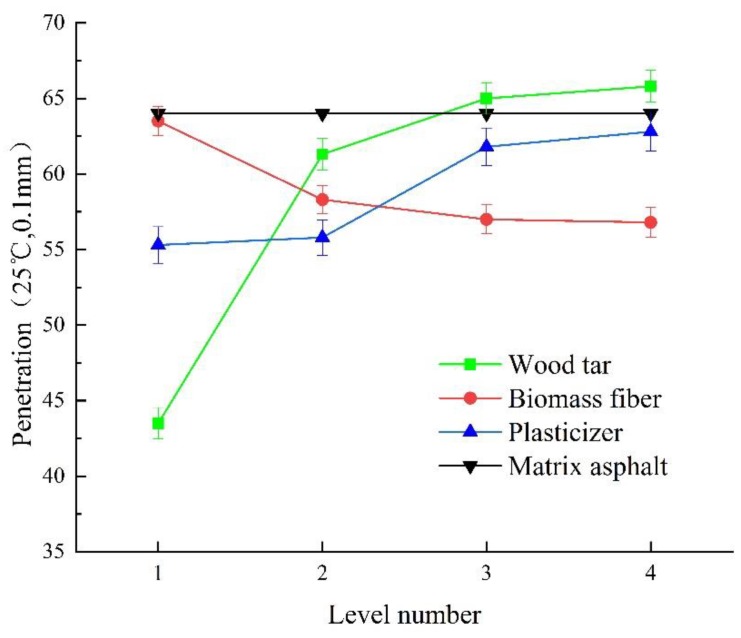
Change rule of penetration of wood tar-based rejuvenated asphalt under different factors and levels.

Figure 5 shows the change rule of penetration of wood tar-based rejuvenated asphalt under different factors and levels.

It can be seen from Figure 5 that (1) with the increase of the content of wood tar, the penetration of rejuvenated asphalt increased. When the content of wood tar increased by 5wt%, the penetration value of rejuvenated asphalt increased by 0.1–1.8 mm. This is because wood tar belongs to oil material, which can be well compatible with asphalt, and the aged asphalt will be softened after being compatible with wood tar [55]. When the content of wood tar is 10 wt%, the slope of change curve of penetration of rejuvenated asphalt is the largest, which means that when the content of wood tar is more than 10 wt%, the growth rate of penetration slows down significantly. Further, 15 wt% wood tar can restore the penetration of aged asphalt to the level of original asphalt.

(2) With the increase of biomass fiber content, the penetration of rejuvenated asphalt decreased gradually. When the content of biomass fiber increased by 0.1 wt%, the penetration value of rejuvenated asphalt decreased by 0.1–0.5 mm. This is because the distribution of the biomass fiber in the rejuvenated asphalt system is uniform and dense, so the whole system is thicker and the penetration is lower. When the content of biomass fiber is 0.3 wt%, the penetration value of rejuvenated asphalt is the closest to that of original asphalt.

(3) With the increase of the content of plasticizer, the penetration of rejuvenated asphalt increased. Because plasticizer as a light component can adjust the proportion of light and heavy component in asphalt. In particular, plasticizer can increase the contents of light components, saturates and aromatics, and then soften the aged asphalt to a certain extent [56]. When the content of plasticizer is 5 wt%, the penetration value of rejuvenated asphalt is close to that of original asphalt, and meets the requirement of the specification (6~8 mm).

(4) By comparing the range of the three factors, it can be seen that the order of factors influencing the penetration of rejuvenated asphalt is A > C > B. Among them, the combination A3B1C4 can make the penetration value of the rejuvenated asphalt reach the level of the original asphalt. That is, the content of wood tar is 15 wt%, the content of biomass fiber is 0.3 wt%, and the content of plasticizer is 5 wt%.

### 3.3. Softening Point

**Figure 6:**
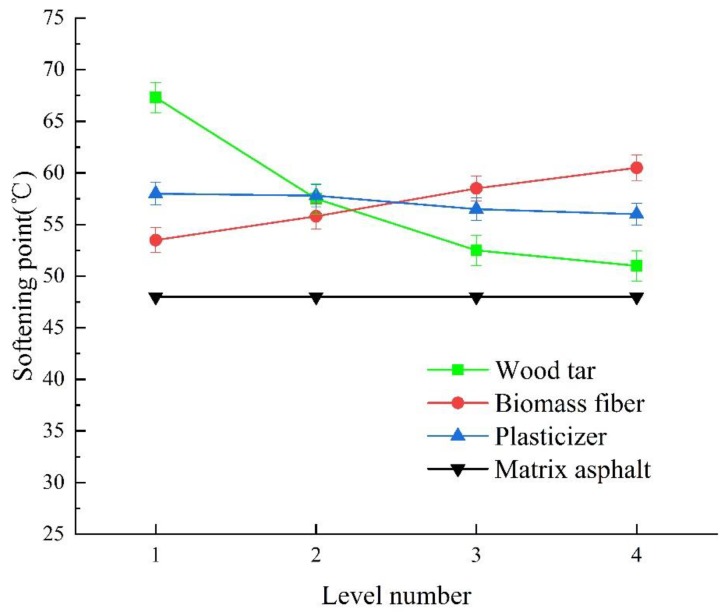
Change rule of softening point of wood tar-based rejuvenated asphalt under different factors and levels.

Figure 6 shows the change rule of softening point of wood tar-based rejuvenated asphalt under different factors and levels.

It can be seen from Figure 6 that (1) with the increase of the content of wood tar, the softening point of rejuvenated asphalt decreased. This is because the wood tar itself has a strong temperature sensitivity, as the temperature rises, the liquidity will be significantly increased, so the softening point of aged asphalt drops [42]. When the content of wood tar increased by 5wt%, the softening point of rejuvenated asphalt decreased by 1.5–10.2 °C. When the content of wood tar is 5wt%, the rejuvenated asphalt has the highest softening point (67.3 °C).

(2) The addition of biomass fiber can improve the softening point of rejuvenated asphalt. This is because the staggered distribution of biomass fiber in asphalt improves the overall stability of asphalt structure. With the increase of biomass fiber content, the internal structure of asphalt will be gradually strengthened, so the temperature sensitivity is weakened and the high-temperature stability is enhanced [34]. When the content of biomass fiber is 0.6 wt%, the rejuvenated asphalt has the highest softening point (60.5 °C).

(3) For every 1wt% increase in plasticizer, the softening point of rejuvenated asphalt decreased by 0.2–1.3 °C. This is related to the oil property of plasticizer itself [57]. When the content of plasticizer is 2 wt%, the rejuvenated asphalt has the highest softening point (58 °C).

(4) By comparing the range of the three factors, it can be seen that the order of factors influencing the softening point of rejuvenated asphalt is A > B > C. The softening point of each rejuvenated asphalt meets the specification requirement (>46 °C). Benchmarked to the softening point of the matrix original asphalt, the optimal ratio of the three additives is A1B1C1. The minimum dosage to meet the requirements of the specification is 5wt% wood tar, 0.3 wt% fiber, and 2 wt% plasticizer.

### 3.4. Ductility

**Figure 7:**
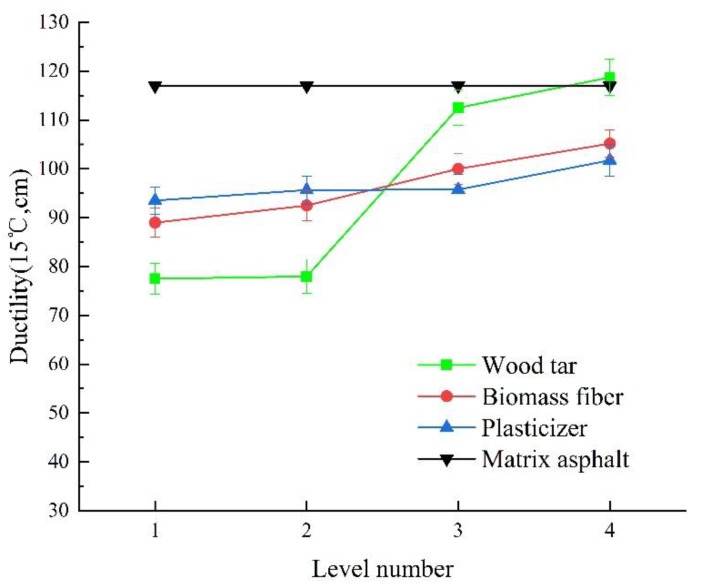
Change rule of ductility of wood tar-based rejuvenated asphalt under different factors and levels.

Figure 7 shows the change rule of ductility of wood tar-based rejuvenated asphalt under different factors and levels.

It can be seen from Figure 7 that (1) since a large amount of light components in wood tar can dilute the heavy components in aged asphalt, the flexibility of rejuvenated asphalt increased with the increase of light components [58,59]. With the increase of the content of wood tar, the ductility of rejuvenated asphalt increased continuously, and when the content of wood tar is 10–15wt%, the ductility value of rejuvenated asphalt increased rapidly. When the content of wood tar increased by 5wt%, the ductility of rejuvenated asphalt increased by 0.5–34.5 mm. When the content of wood tar is 15wt%, the ductility of rejuvenated asphalt is basically similar to that of matrix original asphalt. When the content of wood tar is 20wt%, the ductility of rejuvenated asphalt is slightly higher to that of matrix original asphalt.

(2) Biomass fiber has a positive effect on the ductility of rejuvenated asphalt. After mixing with asphalt, the fiber can fully adsorb with the acid resin in asphalt, sometimes accompanied by chemical action. These effects can make asphalt arranged on the fiber in the form of monomolecular, forming a stable fiber-asphalt composite structure [60]. Meanwhile, the fiber has large surface area, it can absorb lots of oil in wood tar and asphalt and enhance its toughness [61]. Hence, when the fibers in asphalt are pulled by the ductility machine, these fibers can share part of the pull and increase the toughness of asphalt.

(3) With the increase of plasticizer content, the ductility of asphalt increased gradually. This is because plasticizers can weaken the van der Waals force between molecules, so that enhance the mobility between molecules and further strengthen the plasticity of aged asphalt [62]. When the content of plasticizer is 5wt%, the ductility of rejuvenated asphalt reaches the maximum (101.8 cm) and can meet the specification requirement (>100 cm).

(4) By comparing the range of the three factors, it can be seen that the order of factors influencing the ductility of rejuvenated asphalt is A > B > C. The proportion of each factor is A4B4C4. That is, the content of wood tar, biomass fiber and plasticizer is 20 wt%, 0.6 wt%, and 5 wt%, respectively.

### 3.5. Viscosity

**Figure 8:**
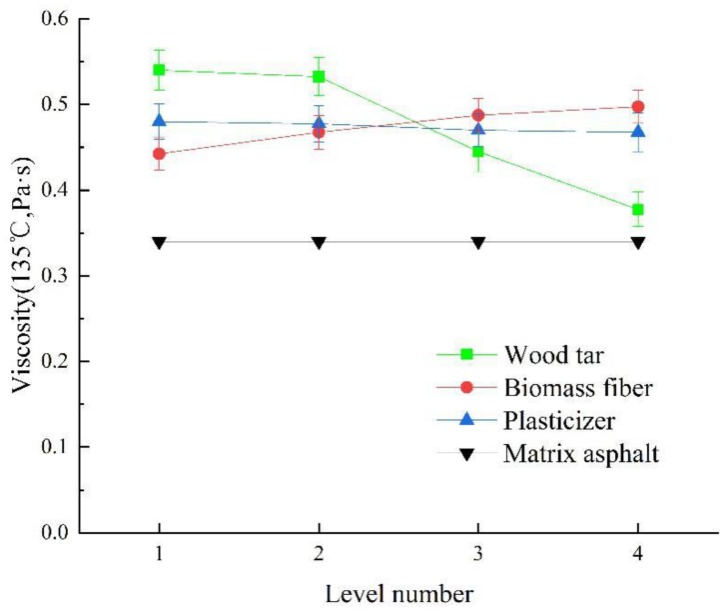
Change rule of viscosity of wood tar-based rejuvenated asphalt under different factors and levels.

Figure 8 shows the change rule of viscosity of wood tar-based rejuvenated asphalt under different factors and levels.

It can be seen from Figure 8 that (1) with the addition of wood tar, the viscosity of rejuvenated asphalt decreased significantly, and the decreasing speed is faster and faster. This is because the viscosity of wood tar is smaller than that of aged asphalt. Due to the good compatibility between wood tar and aged asphalt, wood tar can quickly decrease the viscosity of aged asphalt [63]. When the content of wood tar increased by 5 wt%, the penetration value of rejuvenated asphalt decreased by 0.015–0.0825 Pa·s.

(2) The viscosity of rejuvenated asphalt increased with the increase of content of biomass fiber. The uniform distribution of fibers in asphalt can form a crisscross network structure, which can reduce the fluidity of asphalt and increase the viscosity. When the content of biomass fiber increased by 0.1wt%, the penetration value of rejuvenated asphalt decreased by 0.0125–0.0225 Pa·s.

(3) The viscosity of rejuvenated asphalt decreased with the increase of plasticizer. Because plasticizer can promote the relative motion between macromolecules or chain segments in asphalt, and then play a role of lubricant, so the viscosity value of asphalt decreased and the fluidity becomes better [57].

(4) By comparing the range of the three factors, it can be seen that the order of factors influencing the viscosity of rejuvenated asphalt is A > B > C, and the optimal dosage combination is A3B1C1.

The reason for using oil as the rejuvenator is that oil can supplement the light components required by the aged asphalt, reduce the viscosity of the aged asphalt, and thus achieve the effect of rejuvenation; then through other additives (biomass fiber, plasticizer, compatibilizer, etc.) to further reshape the performance of rejuvenated asphalt to meet the specification requirements [2,64]. To sum up, the optimal dosage combination of wood tar-based rejuvenator is determined as A3B1C4; that is, the content of wood tar, biomass fiber and plasticizer is 15 wt%, 0.3 wt%, and 5 wt% of the mass of aged asphalt respectively. This composition is used in all subsequent studies of wood tar-based rejuvenators.

## 4. Results and Discussion

### 4.1. Physical Properties

The optimal ratio determined by orthogonal test is used to prepare wood tar-based rejuvenator and rejuvenated asphalt. The matrix original asphalt, matrix aged asphalt, wood tar-based rejuvenated asphalt and RA-102# rejuvenated asphalt are labeled as A_O_, A_P_, A_R1_ and A_R2_, respectively. The physical performance test results of each asphalt are shown in Table 6. It can be seen that all indexes in the table meet the requirements of Technical Specifications for Construction of Highway Asphalt Pavement (JTG F40-2004) [65].

It can be seen from Table 6 that (1) the penetration value of A_R1_ is 3.225 times that of A_P_, indicating that wood tar-based rejuvenator can soften aged asphalt and reduce its consistency. The improvement effect of wood tar-based rejuvenator on the penetration of aged asphalt is better than RA-102# rejuvenated asphalt, and the penetration values of the two kinds of rejuvenated asphalt meet the specification requirements.

(2) The softening point of A_R1_ is 28.3 °C lower than that of A_P_, and 9 °C and 2 °C higher than that of A_O_ and A_R2_, respectively. Suggesting that A_R1_ has good high-temperature stability and lower temperature sensitivity. The softening point values of two kinds of rejuvenated asphalt meet the specification requirements.

(3) After aging, the brittleness of asphalt increased noticeably, so the ductility decreased greatly. The ductility value of A_R1_ at 15 °C is 81 cm higher than that of A_P_, indicating that wood tar-based rejuvenator can restore the plasticity and ductility of aged asphalt. Although the ductility value of A_R1_ is less than that of A_O_ and A_R2_, it meets the requirement of specification.

(4) The addition of rejuvenator can effectively decrease the viscosity of aged asphalt. Compared with A_R2_, A_R1_ has better fluidity and workability.

In conclusion, compared with A_O_ and A_R2_, the physical properties of A_R1_ have been restored to meet the requirements of the specification, demonstrating that wood tar-based rejuvenator has excellent rejuvenation performance.

### 4.2. Rheological Properties

#### 4.2.1. High-Temperature Property

**Figure 9:**
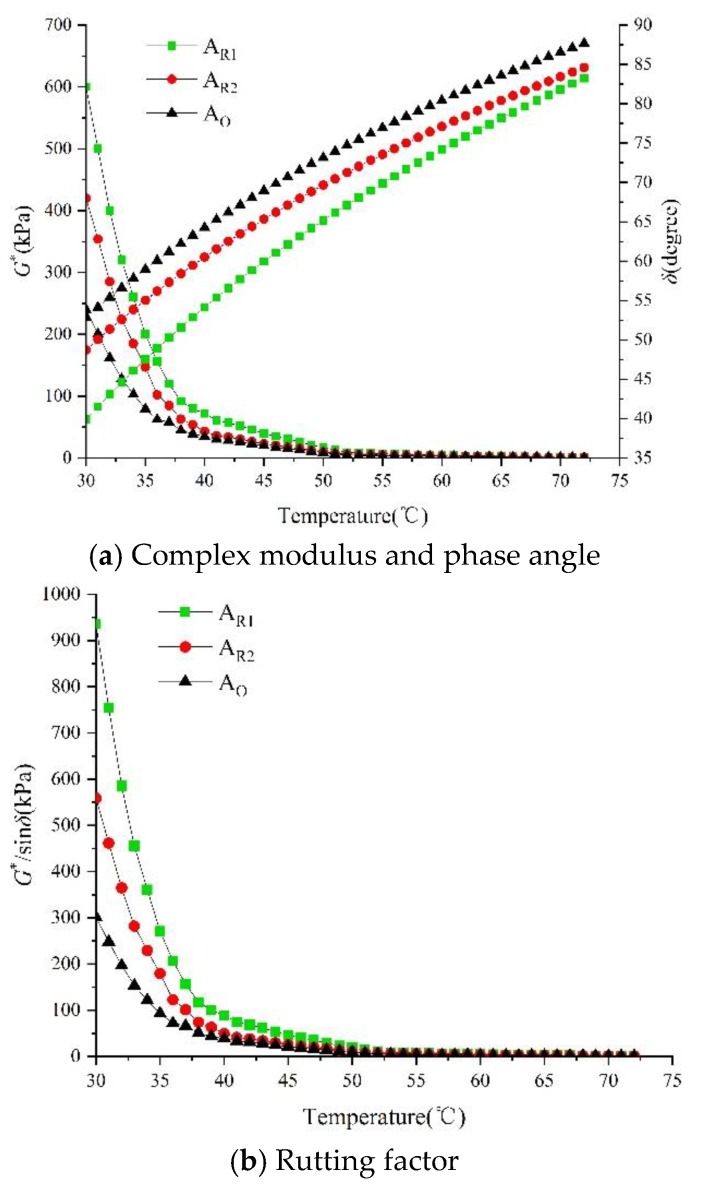
Test results of high-temperature performance of asphalt binder (**a**) G* and *δ*; (**b**) G*/sin*δ*.

The rheological properties of asphalt are related to temperature and time. To characterize the deformation resistance of asphalt at high temperature, DSR is used to measure the complex modulus G* and phase angle *δ* of asphalt at high temperature. The test results are shown in Figure 9.

Complex modulus G* represents the ability of asphalt to resist deformation. The larger the G*, the smaller the deformation of asphalt under high temperature loading, that is, the stronger the deformation resistance of asphalt under high temperature [43]. It can be seen from Figure 9a that the G* value of Ao, A_R1_ and A_R2_ decreased with the increase of temperature, indicating that the increase of temperature can reduce the complex modulus of asphalt and soften the asphalt. At the same temperature, the complex modulus of A_R1_ is the largest, followed by A_R2_, and finally Ao. The results show that the existence of biomass fiber in wood tar-based rejuvenated asphalt can enhance the anti-deformation ability, while RA-102# rejuvenated asphalt and matrix original asphalt are more prone to deformation due to their large amount of light components.

Phase angle *δ* represents the lag between load and deformation of asphalt. The smaller the *δ* value, the better the high-temperature performance of asphalt. On the contrary, the higher the *δ* value, the more obvious the deformation lag effect, the worse the high-temperature performance of asphalt. The *δ* value of the three kinds of asphalt increased with the temperature, indicating that the temperature is positively correlated with the phase angle. At the same temperature, the *δ* value of the matrix original asphalt is the largest, followed by A_R2_ and A_R1_. Therefore, the deformation lag effect of matrix asphalt is the most obvious under the same temperature, and the deformation response of wood tar-based rejuvenated asphalt is the fastest.

Simple G* and *δ* values cannot judge the high-temperature performance of asphalt completely, so the rutting factor G*/sin*δ* is introduced [66]. G*/sin*δ* represents the deformation recovery ability of asphalt. The larger the G*/sin*δ* value, the smaller the unrecoverable cumulative deformation of asphalt, that is, the better the rutting resistance [51]. As can be seen from Figure 9b, the G*/sin*δ* values of the three kinds of asphalt decreased with the increase of temperature. This is because high temperature increases the fluidity of asphalt, which reduces the elastic component of asphalt and thus makes it more viscous, resulting in a large amount of unrecoverable deformation. At the same temperature, the order of G*/sin*δ* values from large to small is A_R1_, A_R2_ and A_O_. Wood tar-based rejuvenated asphalt has a good resistance to cumulative permanent deformation, which is due to the joint action of biomass fiber and plasticizer to enhance the elasticity of wood tar-based rejuvenated asphalt, so that its high-temperature deformation recovery ability.

In conclusion, the high-temperature performance of the two kinds of rejuvenated asphalt is better than that of matrix original asphalt, especially the wood tar-based rejuvenated asphalt. Because of the addition of biomass fiber and plasticizer, the high-temperature resistance to deformation and rutting has been greatly improved.

#### 4.2.2. Low-Temperature Property

**Figure 10:**
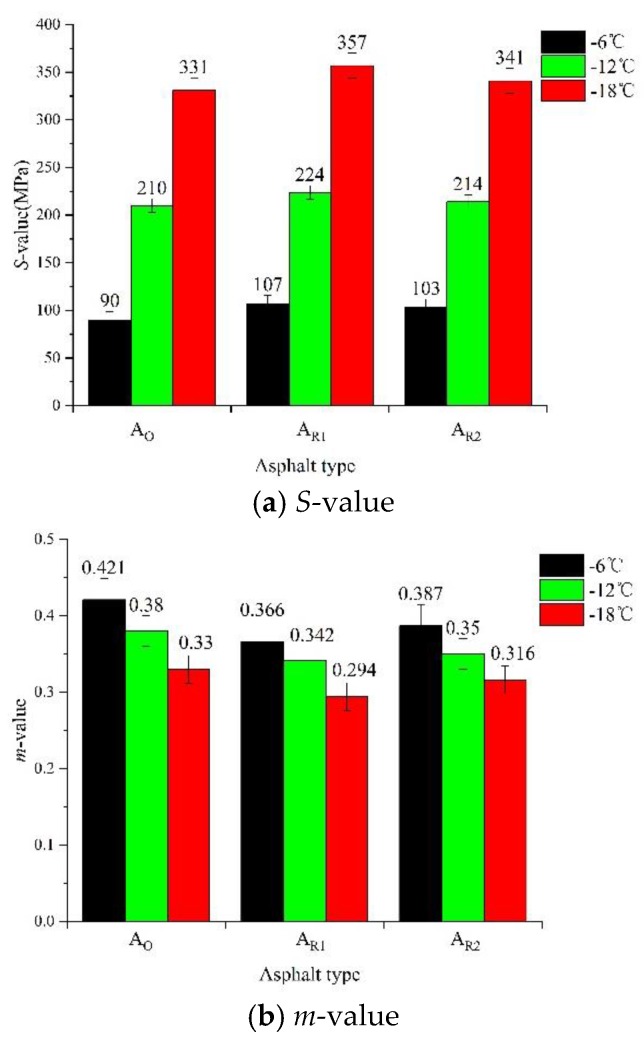
Test results of low-temperature performance of each asphalt (**a**) *S*-value; (**b**) *m*-value.

The low-temperature performance of asphalt binder is evaluated by creep stiffness (*S*-value) and rate of relaxation (*m*-value). The lower the *S*-value and the higher the *m*-value, the better the low-temperature performance of asphalt [38,67]. The test results of low-temperature performance of each asphalt are shown in Figure 10.

The *S*-value of asphalt increased with the decrease of temperature, and the order of *S*-value from large to small is A_R1_ > A_R2_ > Ao. Therefore, both rejuvenators can restore the low-temperature performance of the aged asphalt, but the *S*-value has not been restored to the level of original asphalt. Moreover, the *m*-value of all three kinds of asphalt decreased with the decrease of temperature, and the order of *m*-value is opposite to that of *S*-value. So, the low-temperature performance of matrix original asphalt is better than that of two rejuvenated asphalt.

In addition, the failure performance temperature *T_m_* is defined as the critical temperature when the *m*-value of an asphalt is equal to 0.300 minus 10 °C. The lower the failure temperature, the better the crack resistance of asphalt [68]. The *T_m_* value of Ao, A_R1_ and A_R2_ is −16.71 °C, −13.00 °C and −15.08 °C, respectively. Thus, although wood tar can restore the low-temperature performance of aged asphalt to a certain extent, further research and modification are needed to reach the same level as the original asphalt.

### 4.3. Compositions

#### 4.3.1. Component Analysis

Asphalt contains four main components, including asphaltene, colloid, saturates and aromatics [69]. Both aging and rejuvenation can change the composition of asphalt, so the rejuvenation effect of rejuvenator on aged asphalt can be evaluated by testing the composition of asphalt. The colloidal state of asphalt can be represented by the gel index *I_C_* [45], and the test and calculation results of components of each asphalt are shown in Table 7.

After aging of the original asphalt, the content of asphaltene and colloid increased by 5.29% and 5.91%, while the content of saturates and aromatics decreased by 0.06% and 10.21%, respectively; the *I_C_* value increased from 0.34 to 0.44, indicating that the fluidity of asphalt is greatly reduced, which is consistent with the actual situation. In fact, the alkyl group in the aromatics is connected with the benzene ring, which makes the alkyl group more easily oxidized than the benzene ring under the action of oxygen. After the alkyl is oxidized to polar functional groups (mainly carboxyl groups), polar aromatic hydrocarbons (the main component of colloid) will be formed, which eventually reduce the aromatics content of asphalt after aging [45].

After the rejuvenation of aged asphalt, the content of asphaltene and colloid decreased noticeably, and the content of aromatics increased greatly. This is the opposite of the aging process of asphalt. In other words, the change trend of each component of asphalt during the aging process is the transformation of aromatics to colloid and colloid to asphaltene; while the regeneration is the exact opposite [13]. Compared with A_P_, *I_C_* values of A_R1_ and A_R2_ decreased by 0.04 and 0.05, respectively, and the change rules of each component of the two rejuvenated asphalt are basically the same, indicating that both rejuvenators can significantly change the colloidal structure of asphalt and improve the fluidity of aged asphalt. This is because the main components of the two rejuvenators are light oil, which can soften the colloid and asphaltene in the process of rejuvenating the aged asphalt, thus restore the elastic recovery ability and low-temperature crack resistance of the asphalt [24].

Since RA-102# contains a large number of aromatic components (see Figure 1c), the enhancement effect of A_R1_ on aromatic content of aged asphalt is slightly lower than that of A_R2_. However, wood tar can effectively restore the distribution proportion of components of aged asphalt, and make the aged asphalt change to sol state, so as to have a rejuvenation effect [27].

#### 4.3.2. FTIR

**Figure 11:**
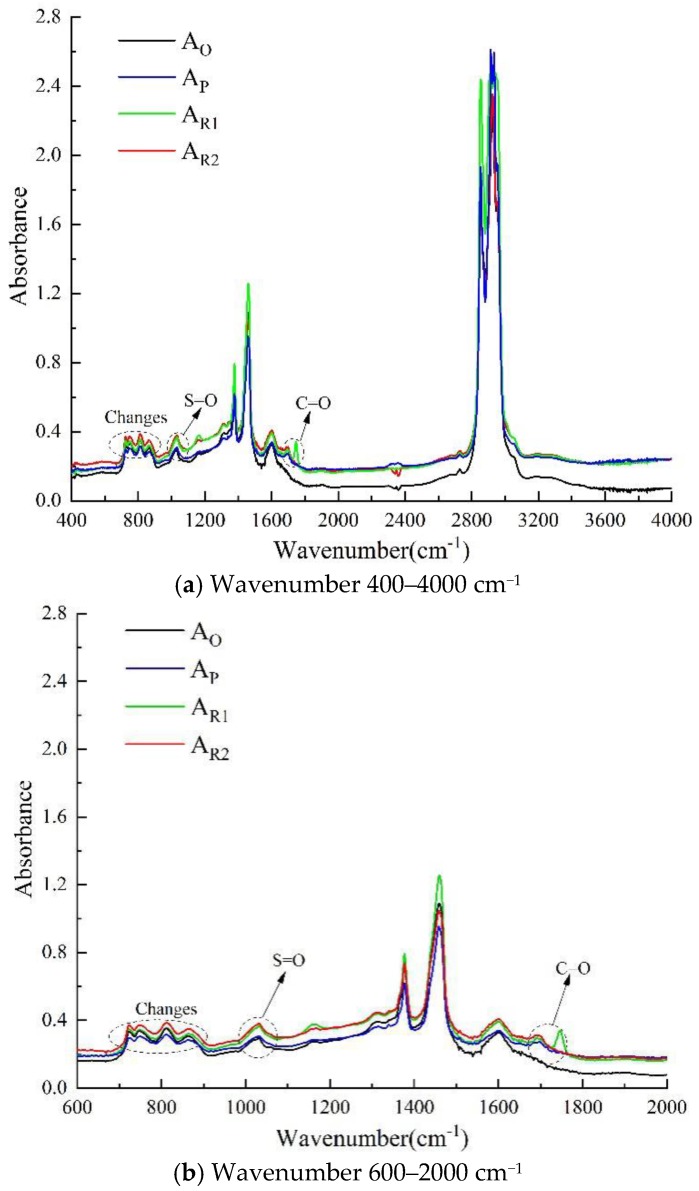
FTIR analysis result of each asphalt binder (**a**) Wavenumber 400–4000 cm^−1^; (**b**) Wavenumber 600–2000 cm^−1^.

**Figure 12:**
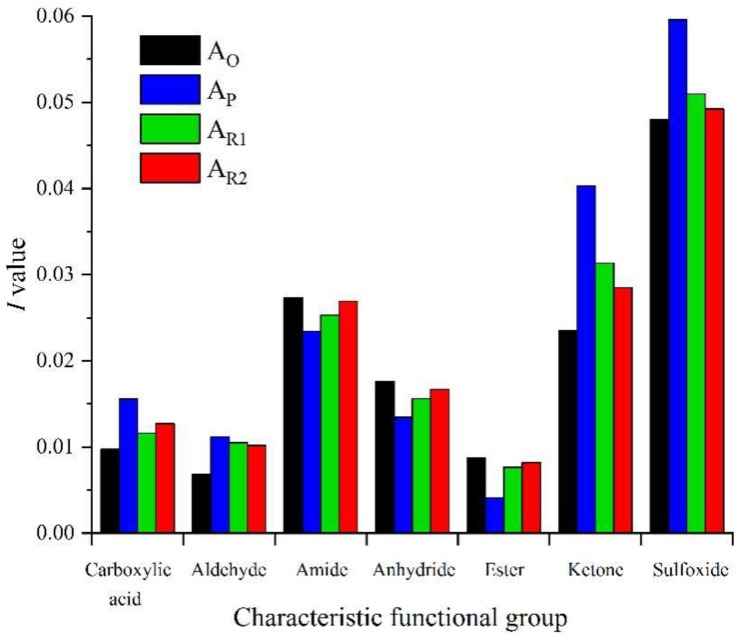
Change rule of aging coefficient of each group.

FTIR was used to analyze the changes of chemical groups before and after asphalt aging and rejuvenation. The results of FTIR analysis are shown in Figure 11 and Figure 12. It can be seen from Figure 11 that after asphalt aging, the absorption peak strength of aromatic components at 720–885 cm^−1^ is weakened, while the absorption peak strength of S=O bond at around 1030 cm^−1^ is enhanced, and a new C=O peak appears at 1700 cm^−1^ of aged asphalt and two kinds of rejuvenated asphalt, indicating that oxidation reaction occurs during asphalt aging [13]. Since there is a C=O absorption peak at 1760 cm^−1^ for wood tar, there is also an obvious C=O absorption peak at 1760 cm^−1^ for wood tar-based rejuvenated asphalt. In addition, the strength of the C-H bond (wavenumber 720–885 cm^−1^) and the N=O bond (wavenumber 1550–1630 cm^−1^) of the two rejuvenated asphalt is obviously enhanced, which shows that the rejuvenator can effectively supplement the light components missing in the aging process of asphalt [46].

As shown in Figure 12, the *I* values of carboxylic acid, aldehyde, ketone, and sulfoxide functional groups increased after asphalt aging, indicating that the viscosity and hardness of asphalt increased, and the rutting resistance of asphalt mixture improved, but the fatigue performance of asphalt mixture was affected [49,50]. The aging effect makes the I values of amide, anhydride and ester of asphalt decreased, demonstrating that the cohesion between asphalt and aggregate decreased, which leads to the decrease of anti-stripping performance of asphalt mixture [51,52]. Moreover, the proportion of aldehyde and ketone in aged asphalt increased the most, showing that these two substances are the main products in the aging process of asphalt. The change of characteristic functional groups of rejuvenated asphalt is just the opposite to that of aged asphalt. Rejuvenation reduced the I values of carboxylic acid, aldehyde, ketone and sulfoxide functional groups of the two aged asphalts, and increased the I values of amide, anhydride and ester functional groups. The results demonstrate that the rejuvenator can reduce the viscosity of asphalt and improve the spalling resistance of asphalt mixture, but slightly reduce the deformation resistance of asphalt at high temperature, which is consistent with the results of viscosity test and high temperature performance test. Among them, the effect of RA-102# rejuvenated asphalt on the above functional groups is slightly stronger than that of wood tar-based rejuvenator.

In conclusion, the addition of wood tar-based rejuvenator resulted in the new absorption peak of C=O at 1760 cm^−1^ in aged asphalt, which is caused by the vibration of C=O bond in wood tar. Besides, the strength of the absorption peak of the aromatic functional group (C-H bond) increased noticeably, while the strength of the absorption peak of the carbonyl (C=O bond) and sulfoxide (S=O bond) decreased greatly. According to the theory of functional group properties [38], after rejuvenation of aged asphalt with wood tar-based rejuvenator, the change of seven characteristic functional groups shows that wood tar can react with aged asphalt chemically, reduce its viscosity, and restore its service performance while adjusting its components.

## 5. Conclusions

In this paper, the composition of wood tar-based asphalt rejuvenator is determined by an orthogonal test. On the basis of clarifying the basic properties of wood tar-based rejuvenated asphalt, the physical properties, rheological properties and components of the matrix original asphalt, matrix aged asphalt, wood tar-based rejuvenated asphalt and RA-102# rejuvenated asphalt are compared and analyzed. Based on the laboratory experiments, the following conclusions can be drawn.

(1) The optimal ratio of wood tar-based rejuvenator is the content of wood tar, biomass fiber, plasticizer, compatibilizer and stabilizer is 15wt%, 0.3wt%, 5wt%, 0.3wt%, and 1wt% of the mass of aged asphalt, respectively;

(2) Wood tar can soften the aged asphalt sufficiently and improve the ductility and plasticity of the aged asphalt. The basic properties of wood tar-based rejuvenated asphalt can be restored to meet the requirements of specification. The deformation resistance of rejuvenated asphalt is higher than that of matrix original asphalt;

(3) Because of the synergism between biomass fiber and plasticizer, wood tar-based rejuvenated asphalt has good resistance to cumulative permanent deformation, but its low-temperature crack resistance needs to be further improved;

(4) Aging transforms the asphalt from sol-state to gel-state, manifesting that the viscosity of asphalt increased and the fluidity decreased. Rejuvenation is the reverse process of aging. Wood tar-based rejuvenator can effectively improve the colloidal structure and fluidity of aged asphalt, thus restoring its workability;

(5) Wood tar-based rejuvenator can react with the aged asphalt and reduce the vibration strength of carbonyl and sulfoxide groups, so as to reduce its viscosity and restore its service performance while adjusting its components;

(6) Through the results of physical properties, rheological properties and compositions, it showed that the road performance, colloidal structure and reaction characteristics of the wood tar-based rejuvenated asphalt are comparable with those of RA-102# rejuvenated asphalt, which makes wood tar-based rejuvenator possess potential qualification for use in the rejuvenation of aged asphalt. However, its low-temperature needs to be further improved.

## 6. Future Work

In general, the service performance of wood tar-based rejuvenated asphalt can basically meet the requirements of the specification, which means that it has the potential to restore the service performance of aged asphalt. However, the test results show that the ductility and low-temperature crack resistance of wood tar-based rejuvenated asphalt need to be improved. The cost and environmental impact of wood tar-based rejuvenated asphalt and its mixture are not yet clear. Therefore, it is necessary to carry out in-depth research and optimization on wood tar-based rejuvenator and rejuvenated asphalt. Specific points to implement include:

(1) Further clarify the impact of wood tar from other biomass sources (such as pine, fir, etc.) on the performance recovery of aged asphalt, and broaden the range of wood tar materials to optimize its rejuvenation effect;
(2) The ductility, low-temperature crack resistance, and fatigue resistance of wood tar-based rejuvenated asphalt can be further improved by introducing appropriate additives (such as SBS);
(3) Explore the molecular structure and chemical composition of wood tar to meet the rejuvenation needs of different types of aged asphalt (matrix asphalt, SBS modified asphalt, etc.);
(4) Evaluate the road performance of wood tar-based rejuvenated asphalt mixture;
(5) Evaluate the energy consumption, cost, and environmental impact in the preparation and use of wood tar-based rejuvenated asphalt and its mixture.

## Figures and Tables

**Table 1:** Basic properties of 70# matrix asphalt.

Property	Unit	70# Matrix Asphalt
Penetration (25 °C)	0.1 mm	64
Penetration index	-	−0.124
Softening point	°C	48
Ductility (15 °C)	cm	117
Viscosity (135 °C)	Pa·s	0.34

**Table 2:** Basic properties of bamboo.

Category		Content (%)	Test Method
Physical property	Moisture	7.0	Dry weightlessness method
	Volatile matter	90.9	
	Fixed carbon	0.2	Slow ashing
	Ash	1.9	
Elementary composition	C	46.90	Elemental analysis
	H	5.84	
	N	0.22	
	S	0.03	
	O_b_	47.01	
Composition	Cellulose	41.0	Van Soest
	Hemicellulose	25.7	
	Lignin	26.1	

**Table 3:** Basic properties of RA-102# rejuvenator.

Property	Measured Value	Specified Value
Viscosity (60 °C, Pa·s)	5370	50–60000
Flash point (°C)	241	≥220
Saturates content (%)	20.3	≤30
Aromatics content (%)	64.2	-
Viscosity ratio before and after TFOT	1.3	≤3
Mass change before and after TFOT (%)	0.5	−3~3

**Table 4:** The wavenumber range and vibration form of each C=O group and its influence on asphalt.

Type	Absorption Peak Location (cm^−1^)	Vibration Form	Effects on Asphalt Properties
Carboxylic acid	1700–1725	Stretching vibration	Rutting resistance [49]
Aldehyde	1720–1740	Stretching vibration, strong intensity	Rutting resistance and fatigue property [50]
Amide	1640–1690	Stretching vibration, strong intensity	Improve adhesion [51]
Anhydride	1740–1775 and 1800–1830	Stretching vibration, two bands	Improve adhesion [51]
Ester	1735–1750	Stretching vibration, strong intensity	Improve adhesion [52]
Ketone	1665–1715	Stretching vibration, strong intensity	Increase the viscosity [47]

**Table 5:** Composition of wood tar-based rejuvenator and performance test results of its rejuvenated asphalt.

Serial Number	Factors and Levels (%)			Test Index			
	Wood Tar	Biomass Fiber	Plasticizer	Penetration (25 °C, 0.1 mm)	Softening Point (°C)	Ductility (15 °C)	Viscosity (135 °C, Pa·s)
S-1	5	0.3	2	43	65	60.0	0.52
S-2	5	0.4	3	42	67	59.3	0.53
S-3	5	0.5	4	46	68	64.2	0.55
S-4	5	0.6	5	43	69	60.1	0.56
S-5	10	0.3	3	60	54	90.1	0.51
S-6	10	0.4	2	59	57	74.1	0.53
S-7	10	0.5	5	62	58	85.7	0.54
S-8	10	0.6	4	64	61	69.8	0.55
S-9	15	0.3	4	73	49	114.0	0.40
S-10	15	0.4	5	68	51	118.3	0.43
S-11	15	0.5	2	59	54	108.4	0.47
S-12	15	0.6	3	60	56	101.4	0.48
S-13	20	0.3	5	78	46	121.2	0.34
S-14	20	0.4	4	64	48	116.0	0.38
S-15	20	0.5	3	61	54	107.3	0.39
S-16	20	0.6	2	60	56	103.2	0.40

**Table 6:** Physical performance test results of each asphalt.

Property	A_O_	A_P_	A_R1_	A_R2_	Specification Requirement
Penetration (25 °C, 0.1 mm)	64	20	64.5	63	60–80
Softening point (°C)	48	85.3	57	55	≥46
Ductility (15 °C, cm)	117	21	102	113	≥100
Viscosity (135 °C, Pa·s)	0.34	0.93	0.42	0.48	-

**Table 7:** Composition and gel index of each asphalt.

Asphalt Type	Composition (%)				*I_C_*
	Asphaltene	Colloid	Saturates	Aromatics	
A_O_	11.01	25.37	14.31	48.35	0.34
A_P_	16.30	31.28	14.25	38.14	0.44
A_R1_	12.79	26.71	15.18	43.25	0.40
A_R2_	12.40	24.37	14.30	44.87	0.39
